# Multimodal imaging supporting the pathophysiology of white dot syndromes

**DOI:** 10.1186/s12348-021-00261-3

**Published:** 2021-09-16

**Authors:** Ilaria Testi, Rocco Luigi Modugno, Carlos Pavesio

**Affiliations:** 1 Department of Uveitis, Moorfields Eye Hospital, National Health Service Foundation Trust, 162 City Rd, Old Street, London, EC1V 2PD UK; 2 Department of Ophthalmology, University of Padova, Padova, Italy

**Keywords:** White dot syndromes, Choriocapillaris, Outer retina, retinal pigment epithelium (RPE), Multimodal imaging, Fundus fluorescein angiography (FFA), indocyanine angiography (ICGA), optical coherence tomography angiography (OCT-A)

## Abstract

White dot syndromes (WDS) represent a heterogeneous group of inflammatory diseases, primarily affecting the outer retina, choriocapillaris and choroid. Recent advances in the field of ocular imaging and development of new technologies, including optical coherence tomography angiography (OCT-A), have allowed a better characterization of the morphology of these conditions. This review will analyse the WDS from an imaging-based perspective, providing a better understanding of the pathophysiology underlying these disorders.

White dot syndromes (WDS) represent a heterogeneous group of inflammatory diseases, primarily affecting the outer retina, choriocapillaris and choroid. WDS include conditions such as multiple evanescent white dot syndrome (MEWDS), punctate inner choroidopathy (PIC), multifocal choroiditis (MFC), acute posterior multifocal placoid pigment epitheliopathy (APMPPE), serpiginous choroiditis and birdshot retinochoroiditis (BRC). The diseases are characterized by a distinctive clinical phenotype derived from the ocular structures involved in the inflammatory process. As in the case of posterior uveitis, multimodal imaging plays a key role in the assessment of a disease, documenting the precise localization of lesions and different levels of tissue involvement. The better characterization of disease morphology, also derived from recent advances in the field of ocular imaging and development of new technologies, has resulted in a better understanding of disease pathophysiology. Multimodal imaging includes several imaging modalities, such as fundus fluorescein angiography (FFA), indocyanine angiography (ICGA), fundus autofluorescence (FAF), optical coherence tomography (OCT) and, more recently, OCT angiography (OCT-A). In addition to providing useful information for characterisation of disease phenotype and diagnosis, these techniques allow evaluation of disease activity, detection of ocular complications and monitoring of response to treatment.

The aim of this review is to analyse the WDS from an imaging-based perspective, providing a better understanding of morphology and pathophysiology of this spectrum of diseases.

## Multiple evanescent white dot syndrome (MEWDS)

**Fig. 1:**
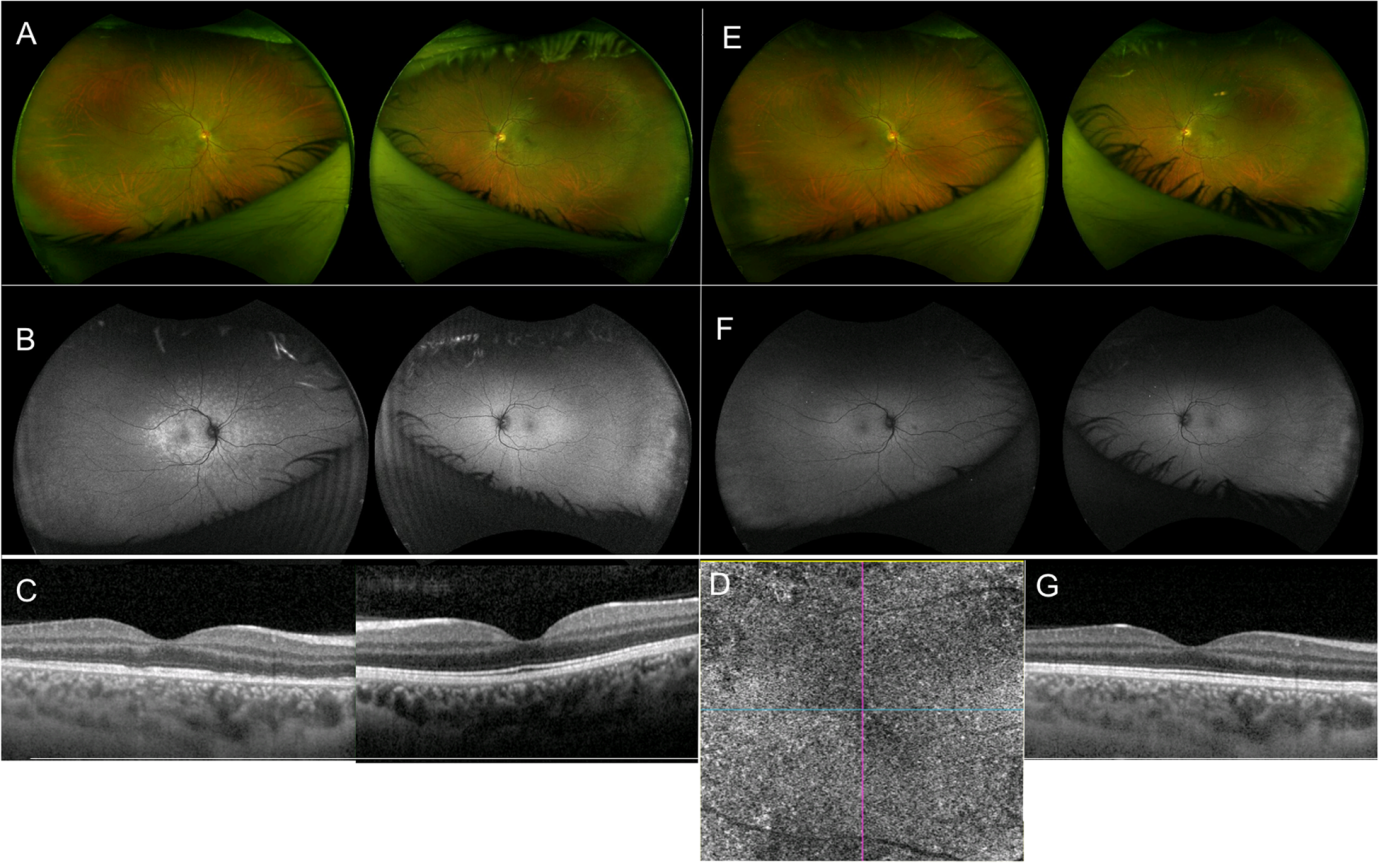
Multiple evanescent white dot syndrome. **A** Right eye ultra-wide field color fundus photograph showing yellowish, deep retinal lesions, located at the posterior pole, extending to mid-periphery; Left eye unremarkable. **B** Right eye ultra-wide field fundus autofluorescence (FAF) showing corresponding hyperautofluoresecent lesions; Left eye unremarkable. **C** Right eye optical coherence tomography (OCT) showing marked disruption of the ellipsoid zone with hypertrophy of the underlying retinal pigment epithelium (RPE); Left eye unremarkable. **D** Right eye optical coherence tomography angiography (OCT-A) showing flow preservation within the choriocapillaris. **E** Right eye ultra-wide field color fundus photograph showing spontaneous resolution of the inflammatory lesions in 4 weeks with no sequelae. **F** Right eye ultra-wide field FAF showing normal fundus autofluoresecence findings. **G** Right eye OCT showing resolution of the outer retina disruption

MEWDS is an idiopathic, acute, unilateral disorder, commonly affecting healthy females in their second to forth decade of life. The disease clinically manifests with yellowish deep retinal lesions, commonly located at the posterior pole and extending to the retinal mid-periphery (Fig. 1A). Mild intraocular inflammation can be present in form of vitritis and/or optic disc oedema. Patients usually complain of sudden and rapid visual loss, dyscromatopsia, photopsias and scotomas. Prodromal flu-like symptoms might precede the ocular manifestations. The disease has a self-limiting course and tends to resolve within weeks with no sequelae and no need for treatment.

MEWDS primary involves the outer retinal layers, including the photoreceptors and the ellipsoid zone, with sparing of the choriocapillaris [1–4]. In the acute phase the lesions appear hyperautofluoresecent on FAF (Fig. 1B). They show characteristic early “wreath-like” hyperfluorescence on FFA, which persists into late frames throughout the exam. Disc hyperfluorescence can be present. On ICGA, lesions typically show late hypofluorescence [5–7]. OCT detects marked disruption of the ellipsoid zone associated with hypertrophy of the underlying retinal pigment epithelium (RPE) **(**Fig. 1C**)**. The lesions involving the ellipsoid zone/RPE correspond to the hyperautofluorescent lesions seen on FAF, the hyperfluorescent lesions detected on FFA and the hypofluorescent spots seen on ICGA, and it is important to note that no areas of flow void are detected on OCT-A (Fig. 1D), demonstrating the sparing of the choroid and, in particular, of the choriocapillaris [1–4]. The hypofluorescent lesions seen in the late frames of ICGA can thus be explained not by choriocapillaris hypoperfusion, but by RPE abnormality secondary to outer retinal involvement, resulting in changes in the indocyanine green uptake by the RPE, which normally determines the physiological late background hyperfluorescence [4]. However, two cases were recently reported, in which OCT-A showed transient areas of flow void at the level of the choriocapillaris [8]. These findings do not establish the choriocapillaris as the primary site of the disease process and could very well represent a secondary response to a RPE dysfunction. Near-infrared fundus autofluorescence (NIR-FAF) show a characteristic foveal “granularity” [9]. It is noteworthy that foveal granularity is primarily appreciated as a uniform and distinguishing clinical feature of MEWDS.

## Acute posterior multifocal Placoid pigment Epitheliopathy (APMPPE) and related placoid disorders

**Fig. 2:**
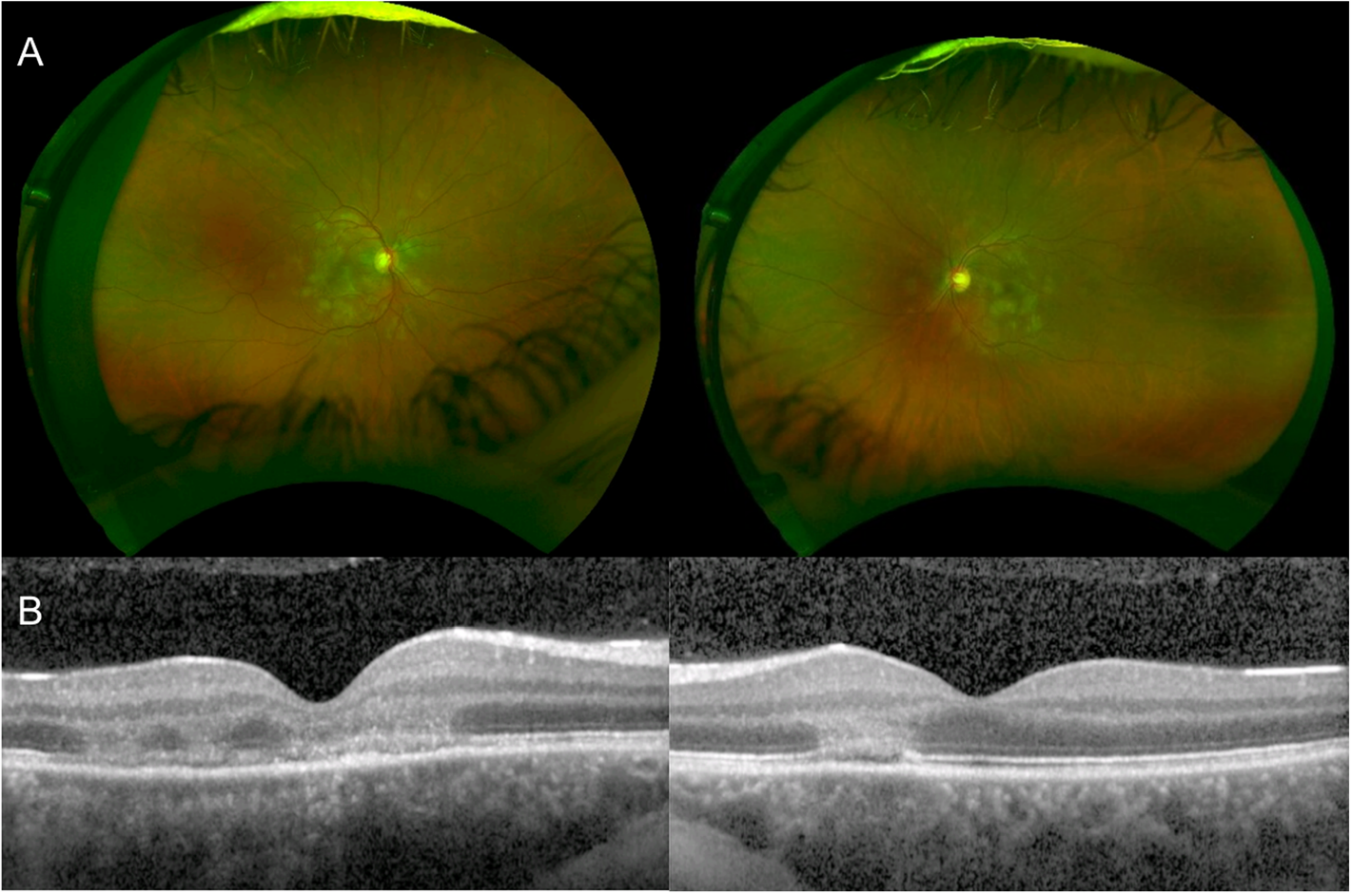
Acute Posterior Multifocal Placoid Pigment Epitheliopathy. **A** Ultra-wide field color fundus photograph of both eyes showing multifocal, yellowish creamy, placoid lesions, located at the posterior pole. **B** Optical coherence tomography (OCT) of both eyes showing disruption of the outer retinal and ellipsoid zone, with hyperreflectivity involving outer nuclear layer with areas of hyperreflectivity appearing in continuity with outer plexiform layer

APMPPE is an idiopathic, acute, commonly bilateral, disorder affecting young healthy adults. The disease clinically manifests with multifocal, yellowish creamy, placoid lesions, located at the posterior pole (Fig. 2A). Patients complain of bilateral visual loss, photopsias and paracentral scotomas. The lesions tend to progressively fade over the weeks, resulting in hyperpigmentation and chorioretinal atrophy, with potential persistent damage to visual function.

**Fig. 3:**
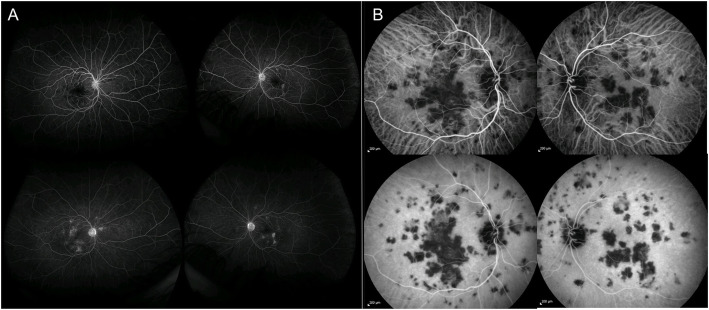
Acute Posterior Multifocal Placoid Pigment Epitheliopathy. **A** Ultra-wide field fluorescein angiography of both eyes showing hypofluorescent lesions in the early phases (right panel, top images), becoming hyperfluorescent in the late frames (right panel, bottom images). **B** Indocyanine angiography of both eyes showing hypofluorescent spots across all phases (left panel, top images: early phases, left panel, bottom images: late phases)

APMPPE is a disease primarily affecting the choriocapillaris and the inner choroid, resulting in secondary changes to the outer retina and RPE [10–13]. The pathophysiology of the disease is supported by the characteristic findings on imaging. APMPPE is characterized by hypofluorescent lesions in the early phases of FFA, becoming hyperfluorescent in the late frames, and hypofluorescent spots across all phases of ICGA (Fig. 3 A,B) [11–13]. On OCT, the lesions appear as disruption of the outer retinal and ellipsoid zone, with hyperreflectivity of the outer retinal layers and RPE (Fig. 2B), resulting in hypoautofluorescent on FAF due to RPE dysfunction/damage [14]. OCT-A demonstrates that such outer retinal changes co-localized with greater areas of decreased flow at the level of choriocapillaris, that in turn correlate with the lesions detected on FFA and ICGA [15, 16]. The findings thus support the theory that APMPPE is a disorder primarily caused by ischemic events occurring at the level of the choriocapillaris and secondary affecting the outer retina and RPE [11, 15, 16]. Area of focal photoreceptor/RPE atrophy can be detected on OCT after resolution of the lesions, with inactive lesions showing hyperfluorescence on FFA, due to window defects derived from RPE atrophy, hypofluorescence on ICGA and diffuse hypoautofluorescence on FAF.

Inner choroid is the primary site of disease pathogenesis in APMPPE related placoid disorders, including persistent placoid maculopathy (PPM) and relentless placoid chorioretinitis (RPC) [16]. PPM is an idiopathic, bilateral chorioretinopathy most commonly affecting adult Caucasian males. The disease clinically manifests with plaque-like, white/yellowish, foveal lesions, characterized by early hypofluorescence and late staining on FFA, and persistent hypofluorescence on ICGA [17]. Choriocapillaris flow reduction appearing as hyposignal on OCT-A, corresponding to the hypoperfusion seen on FFA and ICGA, supports the hypothesis of impaired choroidal vasculature with nonperfusion of the choriocapillaris, compared to alternative leading mechanism of blockage of choroidal fluorescence by inflammatory deposits [16].

RPC, also known as ampiginous choroiditis, is a condition resembling both APMPPE and serpiginous choroiditis, but characterised by atypical retinal distribution. The disease manifests with bilateral, creamy lesions, localized at the level of the RPE. In contrast to APMPPE which is limited to the posterior pole, RPC is characterized by the presence of > 50 to hundreds of lesions, located anteriorly and posteriorly to the equator [18]. The clinical course is characteristically prolonged and relapsing. FFA reveals hypofluorescent lesions in the early phases of FFA with late staining, and hypofluorescent spots across all phases of ICGA. OCT-A provides evidence of flow reduction at the level of the choriocapillaris and outer choroid [16].

In conclusion, OCT-A findings support the hypothesis of primary involvement of the choriocapillaris and inner choroid in APMPPE and related placoid disorders and may help elucidate further the underlying pathogenesis providing valuable information related to the duration and recurrence of choroidal flow reduction associated with this disease spectrum.

## Multifocal choroiditis (MFC) and punctate inner Choroidopathy (PIC)

**Fig. 4:**
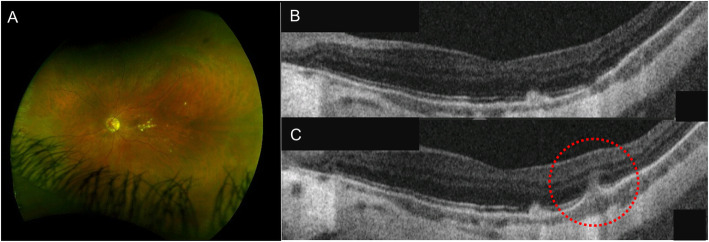
Punctate Inner Choroidopathy. **A** Left eye ultra-wide field fluorescein angiography showing choriorerinal scars located at the posterior pole; **B** correlating with atrophy of the outer retina and retinal pigment epithelium (RPE) on optical coherence tomography (OCT). **C** OCT showing elevation of the RPE with underlying hyporeflective space between the RPE and Bruch’s membrane (circle) and increased penetration of light through the inner choroid, suggestive of disease recurrence

MFC is a chronic, bilateral, inflammatory disorder, manifesting with recurrent episodes of intraocular inflammation, including anterior uveitis and vitritis, associated with yellowish subretinal lesions located at the posterior pole and in periphery. PIC is an idiopathic, inflammatory condition characterized by bilateral, small, discrete and well-defined, yellowish chorioretinal lesions located at the posterior pole, with no associated signs of intraocular inflammation. Both diseases most commonly occur in young patients, especially myopic women in their third to fifth decade of life. Symptoms include scotomas, blurred vision and photopsias. Over time, the inflammatory lesions evolve into chorioretinal scars with variable degree of pigmentation (Fig. 4A). Visual loss might occur due to foveal involvement or development of choroidal neovascularization.

Considering the similarities, MFC and PIC might be considered part of the same disease spectrum [19–22]. Both the diseases involve the choroid, RPE, and outer retina. Findings on OCT scan show elevation of the RPE with sub-RPE material of intermediate reflectivity and surrounding disruption of the outer retina (Fig. 4C) [23, 24]. Active inflammatory lesions appear hypofluorescent in the early phases of FFA with mild late staining, and hypofluorescent on ICGA across all frames [24]. The same lesions manifest with hypoautofluorescent spots with hyperautofluorescent margins on FAF [24]. Once healed, atrophic lesions are characterized by hyperfluorescence as per window defect, with atrophy of the RPE and outer retina on OCT scan **(**Fig. 4B**)** [24]. OCT-A demonstrates well-demarcated areas of reduced flow at the level of the choriocapillaris, co-localizing with active inflammatory lesions corresponding to RPE elevations on OCT scan and hypofluorescent spots on ICGA [25–27]. Whether the changes in the outer retina develop secondary to a primary choroidal involvement or the choriocapillaris is affected following the inflammatory process primarily occurring in the overlying retina has not been elucidated yet.

## Serpiginous choroiditis

Serpiginous choroiditis is a bilateral, chronic, progressive, ocular disorder, manifesting with a characteristic clinical phenotype and course. Greyish subretinal lesions, which start from the peri-papillary region and spread centrifugally, give the disease the characteristic serpiginous pattern. Men are affected slightly more than women. Symptoms include drop in vision, paracentral scotomas, metamorphopsias and photopsias. Over time, active lesions evolve into pigmented chorioretinal scars. However, recurrences occurring weeks to years after the initial episode are common, with reactivation usually manifesting at the border of the atrophic lesions.

The disease is considered to primarily affect the choriocapillaris with secondary involvement of the outer retina and RPE. Active lesions show early hypofluorescence and late hyperfluorescence on FFA, and hypofluorescence across all frames on ICGA [28]. Areas of flow void at the level of the choriocapillaris on OCT-A have been demonstrated to co-localize with the hypofluorescent spots on ICGA, confirming the role of reduced choroidal perfusion in the pathogenesis of the disease [29–31]. OCT in the active phase shows hyper-reflectivity and thickening of the outer retina with disruption of the ellipsoid zone, evolving into decreased retinal thickness and RPE atrophy in the inactive phase [32]. On FAF, active lesions appear hyperautofluorescent, progressively evolving into hypoautofluorescent once healed [33].

## Birdshot retinochoroiditis (BRC)

**Fig. 5:**
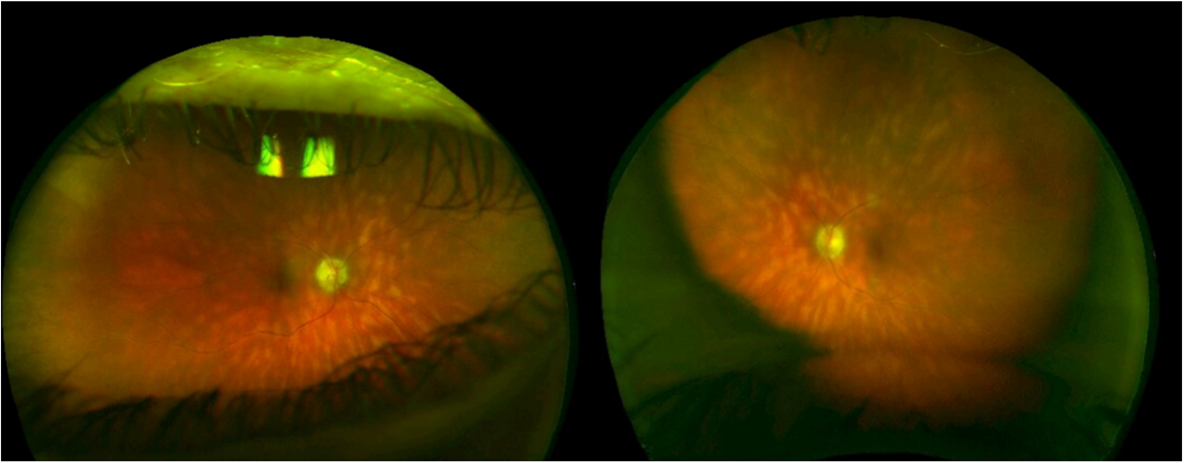
Birdshot retinochoroiditis. Ultra-wide field fluorescein angiography of both eyes showing multiple, yellowish, subretinal lesions, radiating from the optic nerve to the retinal periphery

BRC is a bilateral, progressive, asymmetrical, disease, independently affecting both the retina and the choroid. Retinal involvement is characterized by inflammatory vasculitis, potentially complicated by macular oedema. Choroiditis manifests with multiple, yellowish, subretinal lesions, radiating from the optic nerve to the retina periphery, more concentrated in the infero-temporal area **(**Fig. 5**)** [34, 35]. Middle-aged Caucasian women are characteristically affected by the disorder, complaining of floaters, blurriness, photopsias and decreased vision.

**Fig. 6:**
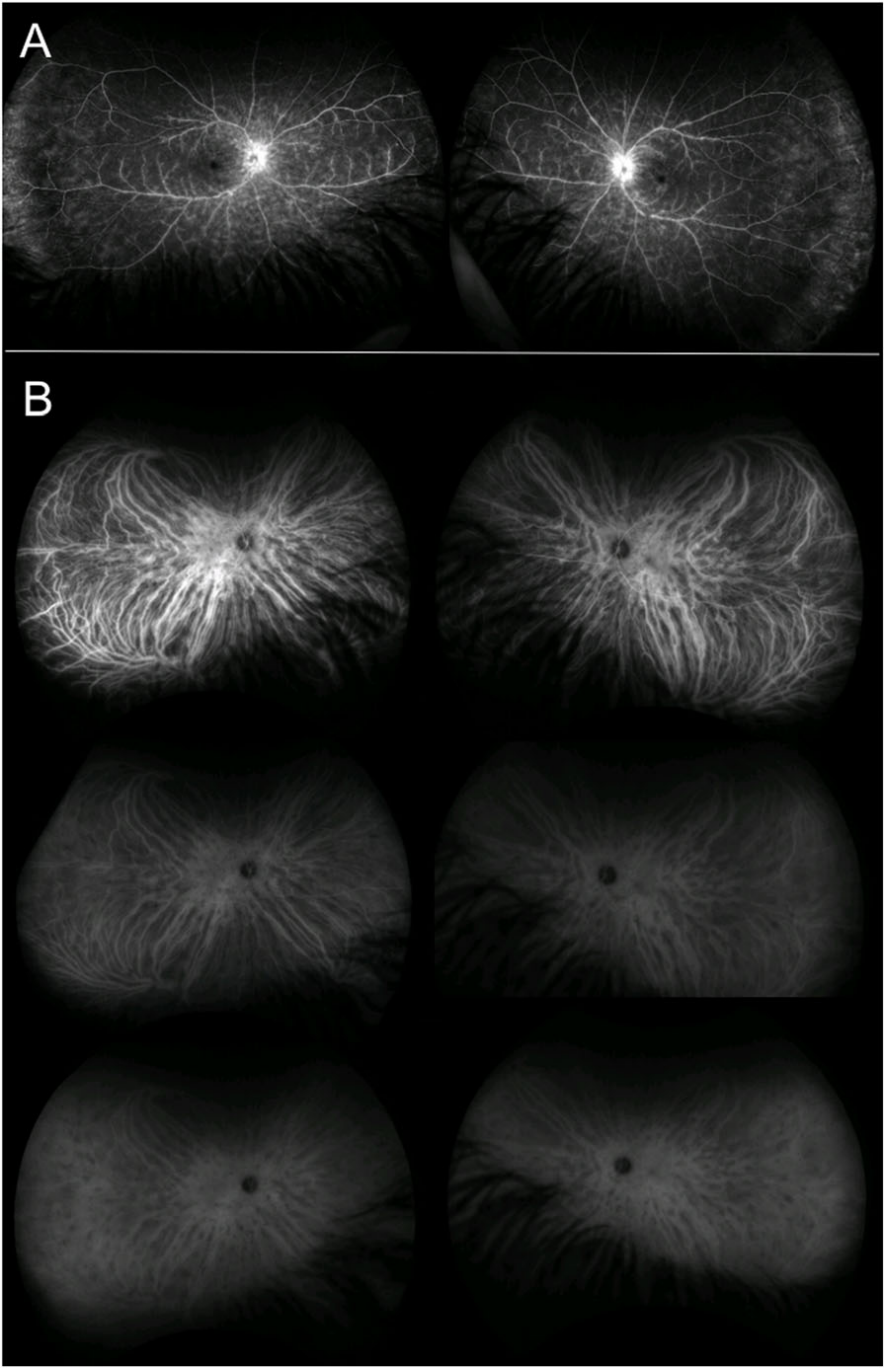
Birdshot retinochoroiditis. **A** Ultra-wide field fluorescein angiography of both eyes showing optic nerve hyperfluorescence and diffuse vascular leakage. **B** Ultra-wide field indocyanine angiography of both eyes showing active choroidal inflammatory lesions appear hypofluorescent throughout the exam, showing extension of the disease from choroidal stroma to choriocapillaris

Retinal involvement is well detected by FFA, which shows optic nerve hyperfluorescence, vascular leakage and macular leakage **(**Fig. 6A**)**. Active choroidal inflammatory lesions appear hypofluorescent in the early phases of FFA with subtle late staining, and hypofluorescent in the early and mid-phases of ICGA, becoming isofluorescent in the late angiographic phase [36, 37]. To support the primary involvement of choroidal stroma in the disease, OCT-A identified areas of flow void in the Haller layer, co-localizing with the hypofluorescent spots on ICGA, with initial sparing of the choriocapillaris [38]. With progression of the disease, the inflammatory damage can extend from the choroidal stroma to the inner choroid, and secondary affects the choriocapillaris and the RPE. At this stage hypofluorescent spots are detected throughout the ICGA **(**Fig. 6B**)** and OCT-A demonstrates full-thickness areas of flow void in the choroid, involving both the stroma and the choriocapillaris.

To summarize, although WDS represent a spectrum of diseases with similar clinical features, they are unique from one another. The name WDS may actually not be the best way of referring to them. Multimodal imaging and recent developments in new technologies, including OCT-A, have provided a better characterization of tissues involvement and disease morphology, allowed their categorization into choroidal syndromes with primary stromal involvement, choriocapillaritis or diseases with primary outer retinal pathology. This has resulted in a better understanding of the pathophysiology at the base of WDS,

## Data Availability

Not applicable.

## Abbreviations

WDS: White dot syndromes
RPE: Retinal pigment epithelium
MEWDS: Multiple evanescent white dot syndrome
PIC: Punctate inner choroidopathy
MFC: Multifocal choroiditis
APMPPE: Acute posterior multifocal placoid pigment epitheliopathy
BRC: Birdshot retinochoroiditis
OCT-A: Optical coherence tomography angiography.
FAF: Fundus autofluorescence
NIR-FAF: Near-infrared fundus autofluorescence
OCT: Optical coherence tomography
FFA: Fundus fluorescein angiography
ICGA: Indocyanine green angiography
